# Evaluation of bone contact area and intercondylar distance changes in orthognathic surgery - a comparison between BSSO and HSSO technique depending on mandibular displacement extent

**DOI:** 10.1007/s00784-024-05584-8

**Published:** 2024-03-01

**Authors:** Stephan Christian Möhlhenrich, Kristian Kniha, Florian Peters, Marius Heitzer, Josef Szalma, Andreas Prescher, Gholamreza Danesh, Frank Hölzle, Ali Modabber

**Affiliations:** 1 Department of Orthodontics, University of Witten/Herdecke, Alfred-Herrhausen Str. 45, 58455 Witten, Germany; 2 Department of Oral and Maxillofacial Surgery, University Hospital of Aachen, Pauwelsstraße 30, 52074 Aachen, Germany; 3 Department Oral and Maxillofacial Surgery, Medical School, University of Pécs, 1. Tüzér St., Pécs, 7623 Hungary; 4 Institute of Molecular and Cellular Anatomy, Medical Faculty of RWTH-Aachen, Pauwelsstraße 30, 52074 Aachen, Germany

**Keywords:** Bilateral sagittal split osteotomy, High oblique sagittal split osteotomy, Orthognathic surgery, Mandible displacement, Bone contact area, Temporomandibular joint

## Abstract

**Objectives:**

The present study aims to assess the impact of bilateral and high oblique sagittal split osteotomy (BSSO/HSSO), as well as displacement distances and directions on the expected and achievable bone contact area (BCA) and changes in the intercondylar distance (ICD). The primary question addressed is whether mandibular splitting through BSSO results in a greater BCA and/or ICD when compared to splitting through HSSO.

**Materials and methods:**

Totally 80 mandibular displacements were performed on 20 fresh cadavers, for each subject, four splints were produces to facilitate mandibular advancement as well as setbacks of 4 and 8 mm. Pre- and postoperative CBCT scans were performed to plan the surgical procedures and to analyze the expected and achieved BCA and ICD.

**Results:**

Regarding the maximum mandibular displacement, the expected BCA for HSSO/BSSO were 352.58 ± 96.55mm^2^ and 1164.00 ± 295.50mm^2^, respectively, after advancement and 349.11 ± 98.42mm^2^ and 1344.70 ± 287.23mm^2^, respectively, after setback. The achieved BCA for HSSO/BSSO were 229.37 ± 75.90mm^2^ and 391.38 ± 189.01mm^2^, respectively, after advancement and 278.03 ± 97.65mm^2^ and 413.52 ± 169.52 mm^2^, respectively after setback. The expected ICD for HSSO/BSSO were 4.51 ± 0.73 mm and 3.25 ± 1.17 mm after advancement and − 5.76 ± 1.07 mm and − 4.28 ± 1.58 mm after setback. The achieved ICD for HSSO/BSSO were 2.07 ± 2.9 mm and 1.7 ± 0.60 mm after advancement and − 2.57 ± 2.78 mm and − 1.28 ± 0.84 mm after setback. Significant differences between the BCA after HSSO and BSSO were at each displacement (*p* < 0.001), except for the achieved BCA after 8-mm setback and advancement (*p* ≥ 0.266). No significant differences were observed regarding ICD, except for the expected ICD after 8-mm setback and advancement (*p* ≤ 0.037).

**Conclusions:**

Compared to the virtual planning, the predictability regarding BCA and ICD was limited. ICD showed smaller clinical changes, BCA decreased significantly in the BSSO group.

**Clinical relevance:**

BCA and ICD might have been less important in choosing the suitable split technique. in orthognathic surgery.

## Introduction

The initial development of the conventional bilateral sagittal split osteotomy (BSSO) technique was credited to Obwegeser [1] and subsequently refined by Dal Pont [2]. This procedure stands as one of the most highly favored surgical approaches for addressing mandibular displacement resulting from skeletal malocclusions of the jaw. Diverse adaptations of the traditional BSSO method, including one put forth by Hunsuck and Epker, have been documented [3, 4]. Additional refinements have addressed factors such as the scope of the osteotomy incision and the chosen surgical instrument [5, 6].

Another method, which is being increasingly employed, is the high oblique sagittal split osteotomy (HSSO) [7]. This technique involves an osteotomy that is strategically positioned higher in relation to the mandibular foramen [8]. In contrast to the conventional BSSO approach, the HSSO technique is anticipated to result in fewer instances of damage to the lower alveolar nerve, a decrease in the extent of the exposed bone surface, and a minimized likelihood of unfavorable splits [9–11]. However, the widespread adoption of HSSO has been hindered by several factors, including the limited bone contact area (BCA) inherent in this technique, which probably leads to delayed bone mending and reduced stability, as well as concerns about the precise alignment of the proximal segment.

HSSO has been comparatively less investigated. Seifert et al. compared both techniques in a retrospective analysis of intra- and postoperative complications over a period of 10 years [12]. They argued that HSSO is a possible alternative to BSSO because newly developed osteosynthesis material significantly reduces the risk of material failure and because BSSO is associated with a higher risk of complications, such as a bad split or sensory disorders. However, BSSO remains the standard for large anterior–posterior displacements of the mandible, probably due to the limited range of mandibular movement that is possible with HSSO. In a previous study, these osteotomy techniques have also been compared in a virtual study setup for mandibular advancements and setbacks up to 10 mm [13]. The mean (standard deviation [SD]) values for the areas of bony surface contact for HSSO and BSSO were 193.94 mm^2^ (63.76) and 967.92 mm^2^ (229.21), respectively, after 10-mm advancement and 202.64 mm^2^ (62.30) and 1108.86 mm^2^ (247.38) after 10-mm setback. However, due to the use of a theoretical study design based on software support, these findings should be interpreted as an approximation of clinical reality. Inhomogeneous intersection surfaces, individual anatomical differences, and/or insufficient split control must be expected to influence the resulting BCA as well as the displacement possibilities.

In this context, the current study aimed to investigate whether mandibular split via BSSO results in a greater BCA and/or larger inner and outer intercondylar distances (ICD and OCD) compared to HSSO, and to assess the impact of displacement distance and direction. In addition, it was checked whether the clinically achieved results corresponded to the theoretically achievable ones.

## Materials and methods

The study design was approved by the ethics committee of the medical faculty at the RWTH Aachen University (Reference: EK 219/16), and institutional authorization was granted by the molecular and cellular anatomy department at the University Hospital of RWTH Aachen, Germany. In this study, 80 mandibular repositions were executed on 20 mandibles, retaining at least one molar dentition in each fresh cadaver head. The cohort consisted of 10 females and 10 males with an age range of 55–85 years and an average age of 70.5 years. In both surgical groups (BSSO and HSSO), each comprising ten subjects, comparable conditions were ensured in terms of missing embalming, dentition status (partially edentulous with end molar), age (BSSO: 72.5 years, HSSO: 68.5 years), and gender (BSSO/HSSO: each 5 females and 5 males), and no other significant differences were observed. Each cadaver head underwent two mandibular advancements and two mandibular setbacks by 4 and 8 mm respectively. For this purpose, four splints were created for each subject to facilitate each mandibular displacement.

### Surgical planning

Prior to the surgical procedures, all subjects were scanned using Galileos CBCT (Sirona, Bensheim, Germany) while in maximal intercuspidation. Additionally, super-hard plaster models of the maxilla were created using Alpenrock (Amann Girrbach, Koblach, Austria) based on impressions produced using Impregum Penta (3 M ESPE, Neuss, Germany). The physical models were then digitized into virtual representations using an orthoX scan 3D model scanner (Dentaurum, Ispringen, Germany). Next, the CBCT scans and the virtual models were converted into the Digital Imaging and Communications in Medicine (DICOM) format and imported into Dolphin 3D Surgery software (Dolphin Imaging & Management Solutions, Chatsworth, CA, USA).

**Fig. 1:**
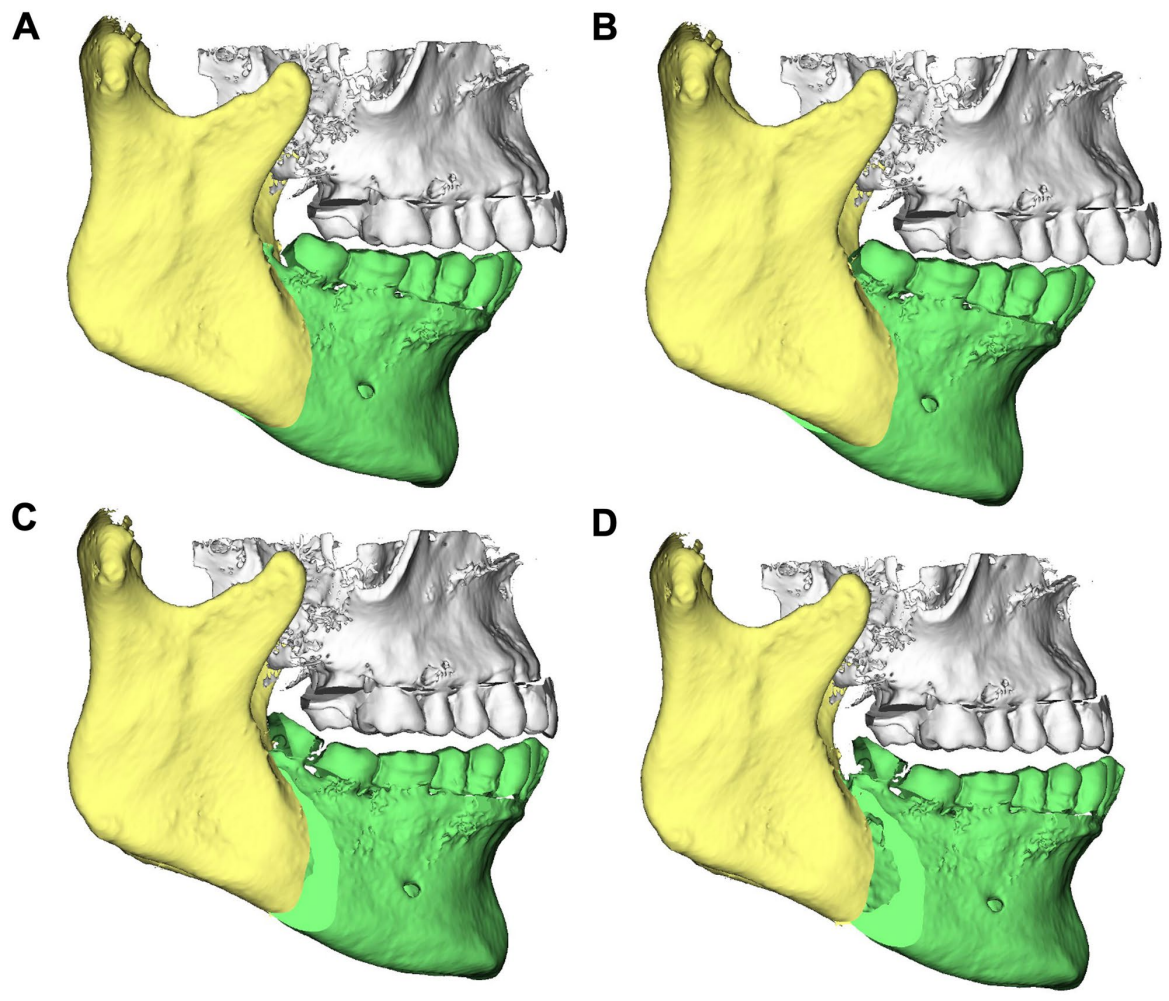
Digital planning of mandibular displacement based on BSSO for (**A**) the -4-mm setback, (**B**) the -8-mm setback, (**C**) the 4-mm advancement, and (**D**) the 8-mm advancement

**Fig. 2:**
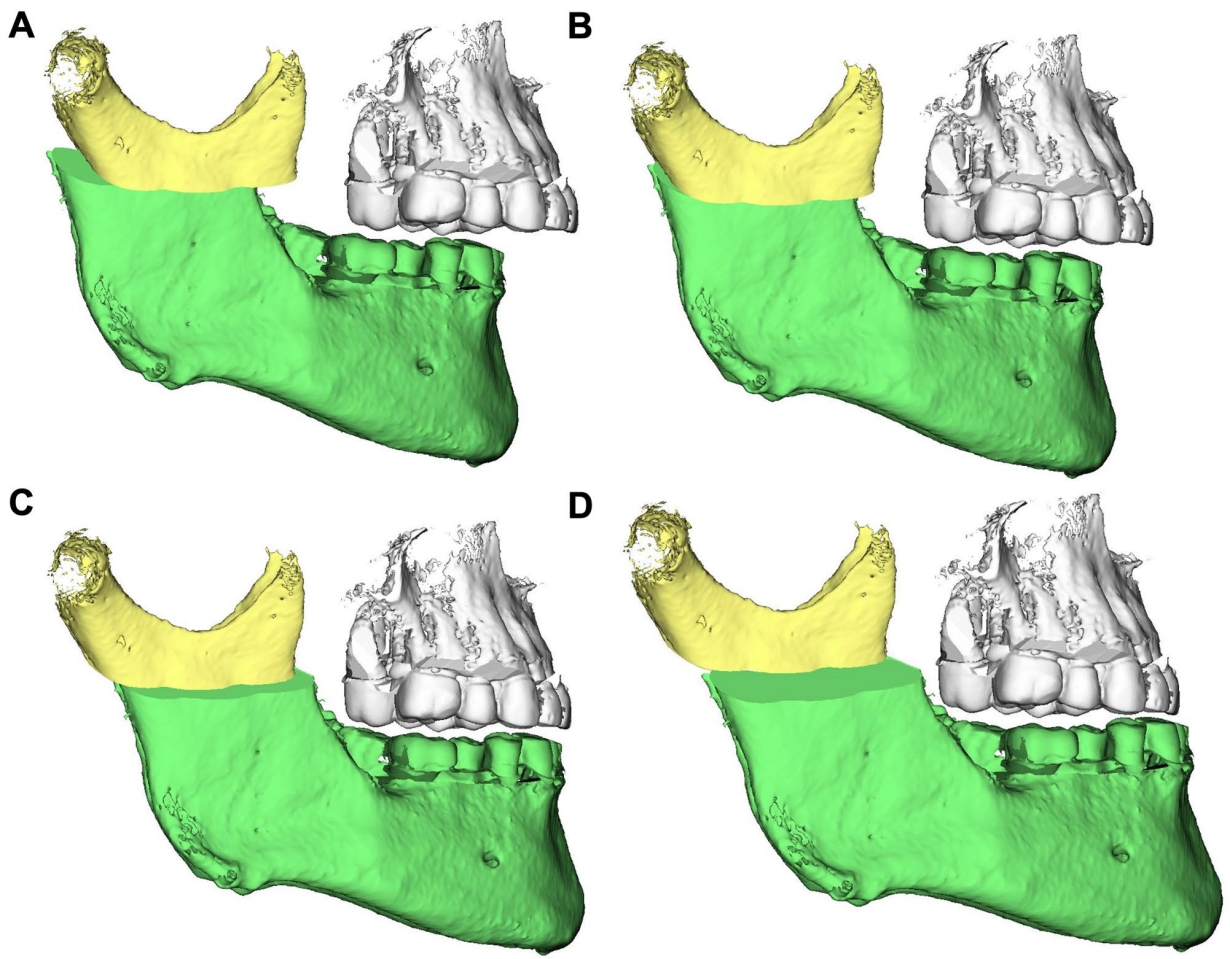
Digital planning of mandibular displacement based on HSSO for (**A**) the -4-mm setback, (**B**) the -8-mm setback, (**C**) the 4-mm advancement, and (**D**) the 8-mm advancement

3D segmentations of the maxilla and mandible were generated by aligning the model casts with the upper and lower jaws, and precise osteotomy lines were delineated. Subsequently, 3D surgical treatments were simulated on each cadaver head. Specifically, four displacements involving mandibular advancements and setbacks of 4 and 8 mm were conducted for each head. Orthognathic surgical interventions using the conventional BSSO technique were carried out on 10 cadaver heads, while the modified HSSO method was used on the other 10 (Figs.  1 and 2). Four custom surgical splints with a vertical occlusion opening of approximately 3 mm were created for each displacement distance using a Form 2 3D printer (Formlabs, Somerville, MA, USA) for each subject.

### Surgical techniques

In the BSSO group, the lingual osteotomy adhered to the Hunsuck/Epker technique, occurring just posterior to the mandibular foramen through the cortical bone by the use of a Lindemann conventional burr drill (Hager & Meisinger GmbH, Neuss, Germany). In addition, a buccal osteotomy was performed through the cortical bone situated between the first and second molars. These osteotomies were combined by a third along the oblique line. Next, the proximal and distal segments were methodically separated through consistent spreading using a spreader and a chisel.

In the HSSO group, the osteotomies were carried out using a GC615R reciprocating saw (Microspeed Aesculap AG, Tuttlingen, Germany). This bone cut was initiated on the lingual side of the ascending ramus, roughly 3 mm above the mandibular foramen, and extended downward to the vestibular side of the ascending ramus.

### Mandibular displacement

For each subject, four splints were produced to facilitate mandibular advancement as well as setbacks of 4 mm an 8 mm. After achieving intermaxillary fixation (IMF) onto the surgical splint using IMF screws and metal wires, osteosynthesis procedures were conducted using Modus 2.0 fixation plates (M-4051 C for BSSO and M-4055 C for HSSO) and M-5243.07 C/4 Modus 2.0 screws (Medartis GmbH, Umkirch, Germany).

### Software measurements

Postoperative CBCT scans were obtained after each displacement. A set of four splints was used, and the process of IMF and osteosynthesis was repeated for each updated configuration.

**Fig. 3:**
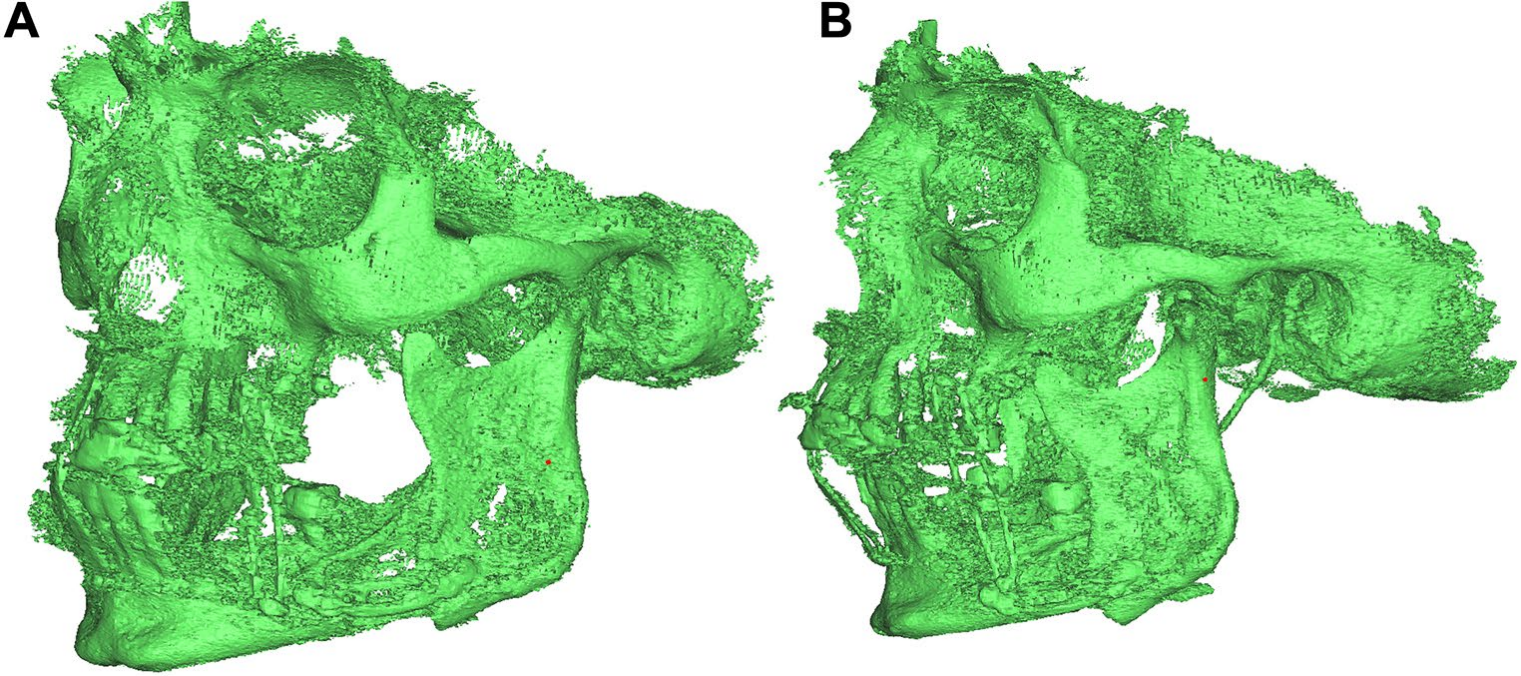
Preliminary bone segmentation of a human cadaver skull characterized by HU in the BSSO group after 8-mm (**A**) advancement and (**B**) setback. The surgical splint was not radiopaque

**Fig. 4:**
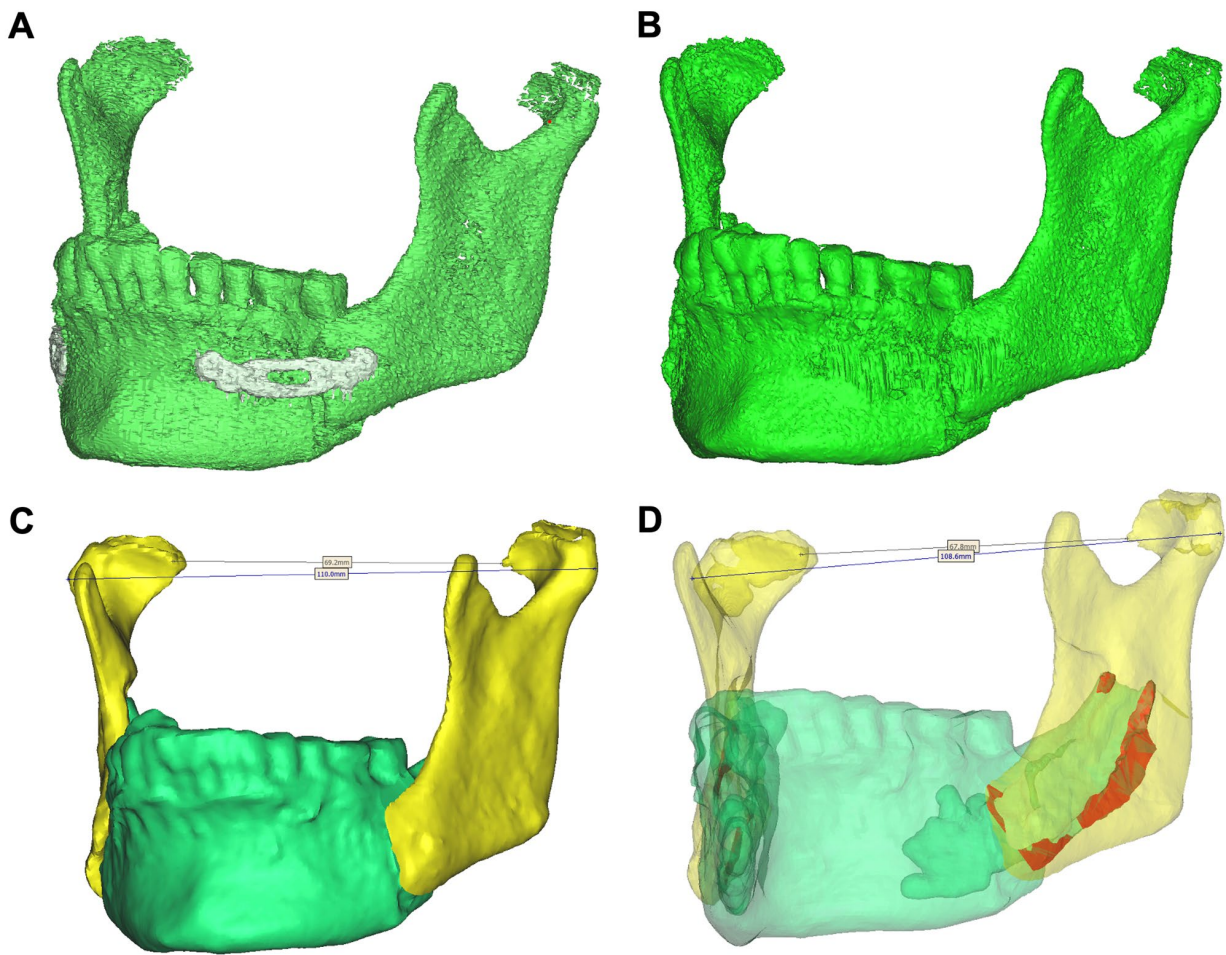
Segmentation analysis process for BSSO. (**A**) Final segmented mandible characterized by HU after the 4-mm advancement. (**B**) Segmented mandible after extracting the osteosynthesis plates and before virtual separation of the segments. (**C**) Compression model of the virtual mandible model and separation of the proximal (yellow) and distal (green) segments. (**D**) Surface contact area and intercondylar distance between the bony segments

**Fig. 5:**
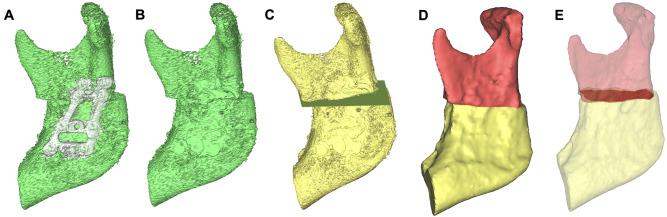
Segmentation analysis process for HSSO. (**A**) Final segmented mandibular angle characterized by HU after the 8-mm setback. (**B**) Segmented mandibular angle after extracting the osteosynthesis plates. (**C**) Separation of the segments by a virtual 0.1-mm osteotomy plane. (**D**) Compression model of the proximal (red) and distal (yellow) segments. (**E**) Surface contact area between the bony segments

The postoperative CBCT data were formatted according to the DICOM standard and imported into ProPlan CMF 3.0.1 software (Materialise, Leuven, Belgium). This software has a positive reputation in the field of computer-assisted jaw surgery due to its marked precision [14, 15]. A bone mask was achieved with Hounsfield units (HU) between 250 and 3000, followed by segmentation with the CMF 3.0.1 software (Fig. 3), and finally virtual models of the distal and proximal mandibular segments were constructed and at the bone contact area digitally superimposed (Figs. 4 and 5). Furthermore, the change (Δ: T1-T0) in postoperative and preoperative maximum inner and outer intercondylar distances were automatically calculated by a single researcher using ProPlan CMF 3.0.1 software. At each displacement, the BCA as assessed twice — once for each side of the ascending ramus. Consequently, 20 bony segment superimpositions were achieved with regard to the respective displacement and surgical techniques (i.e., 2 types of sagittal split osteotomy, 4 displacement distances, and 10 individuals per group).

Furthermore, to obtain the corresponding theoretical achievable BCA and ICD/OCD values, the same procedures were conducted with the datasets of the previous digital planning of the virtual mandibular displacements.

### Statistical analysis

Statistical analyses were made with the aid of Prism (version 10, GraphPad Software Inc., La Jolla, CA, USA). One researcher repeated all measurements after a one-week interval. Calibration was evaluated using the intraclass correlation coefficient (ICC), which consistently yielded values exceeding 0.85 across all variables. In fact, the ICC scores ranged from 0.91 to 0.96, indicating a high level of agreement. The Shapiro-Wilk test was used to assess the normality of variance. Although the BCA values had a normal distribution, this was not the case for all of the measurements related to ICD and OCD. Consequently, the BCA values were subjected to a one-way analysis of variance, followed by a post-hoc Tukey test for multiple comparisons. The intercondylar distance measurements were analyzed using the unpaired nonparametric Mann-Whitney test, which compared the ranks. The threshold for significance was established at *p* ≤ 0.05. All findings are presented as mean values accompanied by their respective SD values.

## Results

**Fig. 6:**
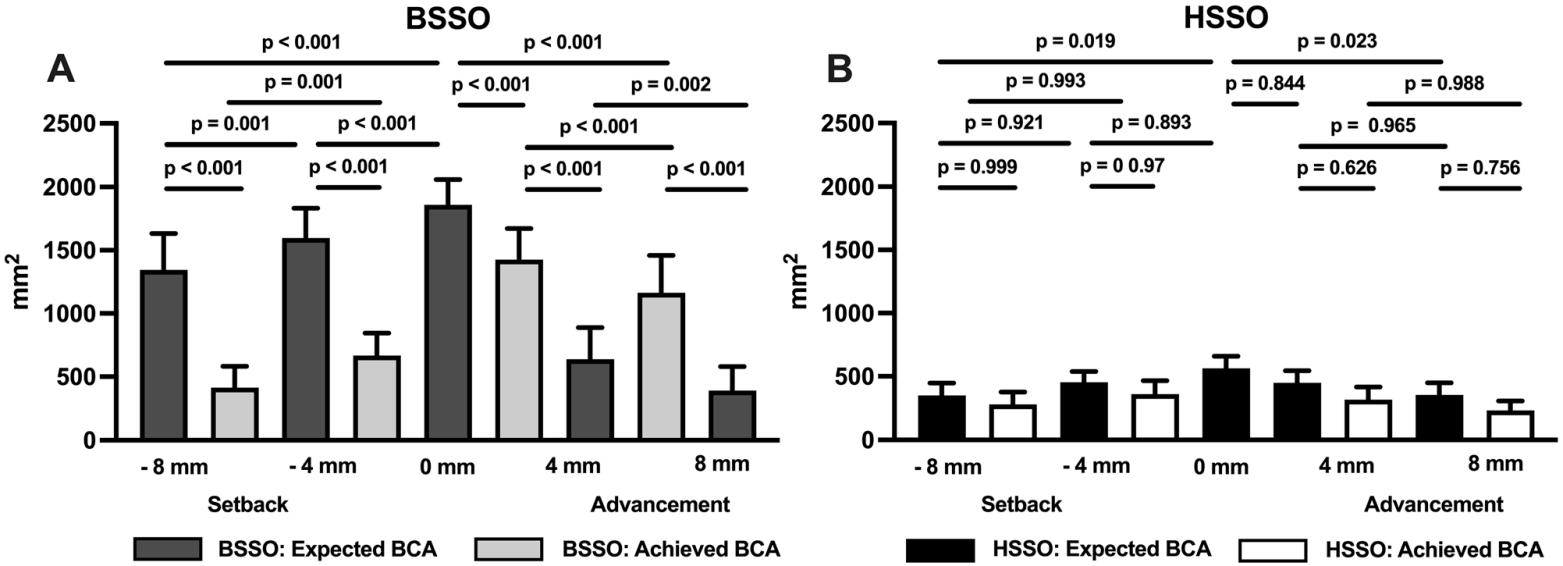
Bar charts displaying mean values (SD) and p-values for statistical comparisons of the expected and achieved bone surface contact area between the segments after the 4-mm and 8-mm mandibular advancement and setback of both rami of the mandibles in the BSSO group (n = 10) and the HSSO group (n = 10)

**Fig. 7:**
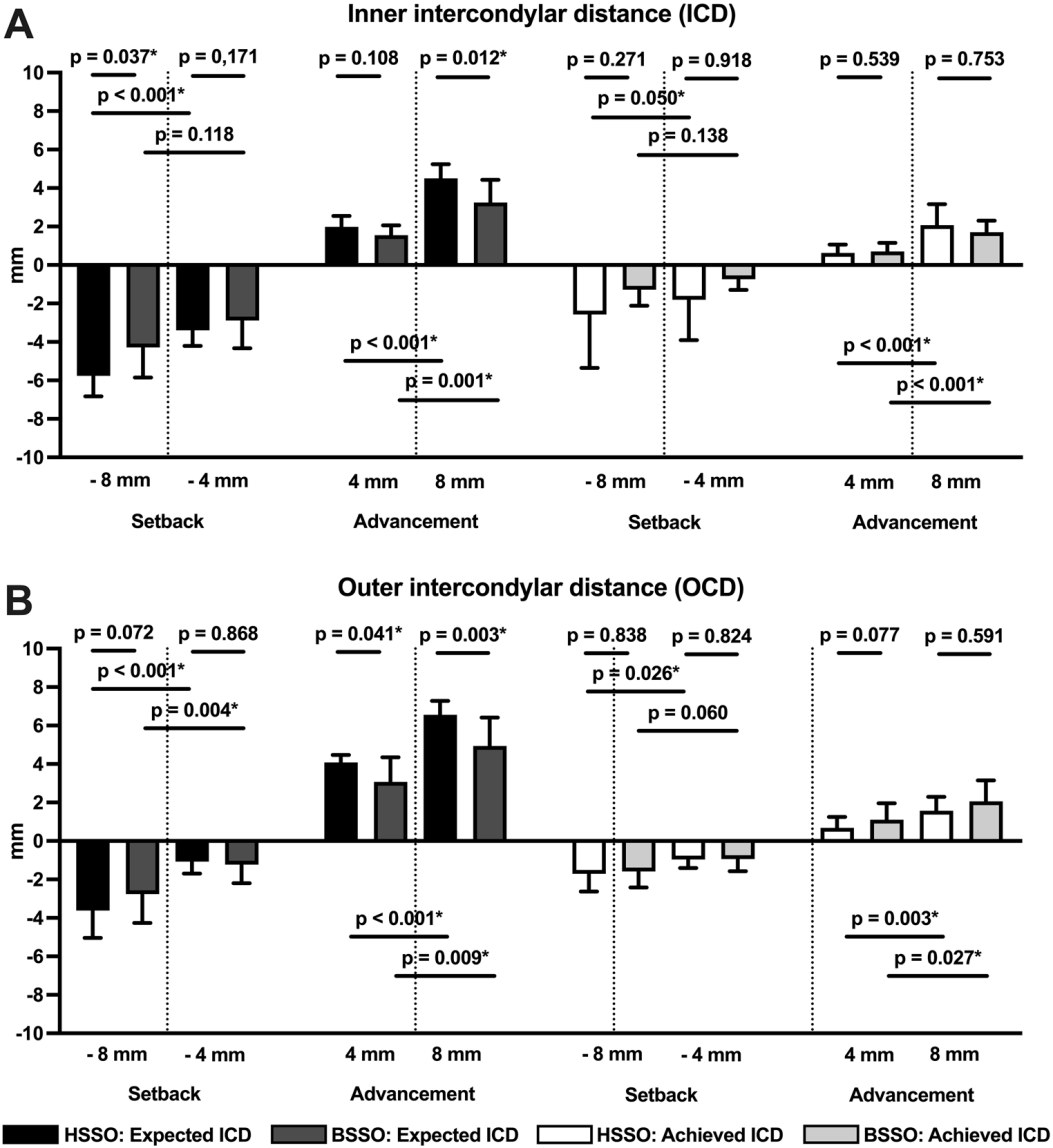
Bar charts displaying mean values (SD) and p-values for statistical comparisons of the expected and achieved ICD and OCD after the 4-mm and 8-mm mandibular advancement and setback in the BSSO group (n = 10) and the HSSO group (n = 10)

The outcomes for the HSSO and BSSO groups, including the *p*-values for the comparisons of the expected and achieved BCAs as well as the ICDs ad OCDs between the bone segments, are presented in Tables 1 and 2. These findings are further illustrated in the corresponding boxplots depicted in Figs. 6 and 7.

**Table 1:** Mean values (SD) of the expected and achieved BCA and corresponding *p*-values for the BSSO group (*n* = 20) and the HSSO group (*n* = 20) based on displacement distances and directions of both rami of the mandibles

					HSSO							BSSO						
	Displacement			%	Mean	SD	Range	Min	Max		%	Mean	SD	Range	Min	Max		p-Value
Reference		0 mm		100	563.24	96.18	342.29	349.41	691.70		100	1859.60	199.85	702.01	1504.50	2206.50		< 0.001
Expected	Setback	− 8 mm		62.0	349.11	98.42	347.62	165.00	512.62		72.3	1344.70	287.23	908.99	871.77	1780.80		< 0.001
		− 4 mm		80.6	454.08	83.94	305.32	272.40	577.72		85.8	1595.90	237.34	1010.30	1049.30	2059.60		< 0.001
	Advancement	4 mm		79.6	448.34	96.78	363.85	232.27	596.12		76.7	1425.60	246.52	775.78	1015.30	1791.00		< 0.001
		8 mm		62.6	352.58	96.55	383.49	130.78	514.27		62.6	1164.00	295.50	1045.60	693.07	1738.70		< 0.001
Achieved	Setback	− 8 mm		49.4	278.03	97.65	323.98	101.36	425.34		22.2	413.52	169.52	633.67	199.33	833.00		0.598
		− 4 mm		63.9	360.07	106.42	402.34	171.01	573.35		35.9	667.21	178.39	714.32	408.82	1123.10		< 0.001
	Advancement	4 mm		55.9	314.92	101.10	424.23	138.60	562.83		34.3	638.27	250.45	872.35	120.63	992.98		< 0.001
		8 mm		40.7	229.37	75.90	233.59	121.96	355.55		21.0	391.38	189.01	593.86	93.46	687.32		0.266

**Table 2:** Mean values (SD) of the expected and achieved ICD and OCD and corresponding *p*-values for the BSSO group (*n* = 10) and the HSSO group (*n* = 10) depending on displacement distances and directions

Split technique	Intercondylar distance	Displacement			Expected						Achieved						
					Mean	SD	Range	Min	Max		Mean	SD	Range	Min	Max		p-Value
HSSO	ICD	Setback	− 8 mm		-5.76	1.07	3.7	-8.2	-4.5		-2.57	2.78	9.8	-10.3	-0.5		0.013*
			− 4 mm		-3.39	0.9	3.3	-5.4	-2.1		-1.81	2.10	7.2	-0.3	-7.5		0.003*
		Advancement	4 mm		1.98	0.57	1.8	0.9	2.7		0.63	0.44	1.4	0.2	1.6		< 0.001*
			8 mm		4.51	0.73	2.4	3.7	6.1		2.07	1.09	2.9	0.9	3.8		< 0.001*
	OCD	Setback	− 8 mm		-3.62	1.42	5.3	-7.2	-1.9		-1.71	0.92	2.7	-3.5	-0.8		0.001*
			− 4 mm		-1.07	0.63	2.0	-0.3	-2.3		-0.96	0.44	1.5	-0.3	-1.8		0.926
		Advancement	4 mm		4.08	0.39	1.2	3.6	4.8		0.68	0.57	1.7	0.1	1.8		< 0.00*1
			8 mm		6.56	0.72	2.3	5.3	7.6		1.57	0.72	1.9	0.6	2.5		< 0.001*
BSSO	ICD	Setback	− 8 mm		-4.28	1.58	4.1	-6.4	-2.3		-1.28	0.84	2.5	-0.2	-2.7		< 0.001*
			− 4 mm		-2.88	1.44	4.7	-5.7	-1.0		-0.74	0.56	1.6	0.0	-1.6		0.002*
		Advancement	4 mm		1.55	0.52	1.7	0.8	2.5		0.71	0.44	1.3	0.0	1.3		0.005*
			8 mm		3.25	1.17	4.1	1.2	5.3		1.7	0.60	1.8	0.4	2.2		0.004*
	OCD	Setback	− 8 mm		-2.76	1.5	5.4	-1.0	-6.4		-1.58	0.84	2.4	-0.5	-2.9		0.041*
			− 4 mm		-1.23	0.97	3.3	-0.5	-3.8		-0.94	0.63	1.7	-0.2	-1.9		0.541
		Advancement	4 mm		3.07	1.28	4.0	0.8	4.8		1.11	0.85	3.0	0.2	3.2		0.002*
			8 mm		4.94	1.47	4.4	1.9	6.3		2.06	1.09	3.4	0.5	3.9		0.003*

* Statistically significant

Regarding the expected BCA, significant differences between the BSSO and HSSO groups were found for all displacement scenarios (*p* < 0.001) (Table 1). However, regarding the achieved BCA, no significant differences were found for the 8-mm setback (BSSO: 413.52 ± 169.52 mm^2^ vs. HSSO: 278.03 ± 97.65 mm^2^, *p* = 0.598) or the 8-mm advancement (BSSO: 391.38 ± 189.01 mm^2^ vs. HSSO: 229.37 ± 75.90 mm^2^, *p* = 0.266).

In the BSSO group, there were significant decreases in the expected and achieved BCA after all of the mandibular advancements and setbacks (*p* ≤ 0.002) (Fig. 6). Compared to the reference BCA of 1859.60 ± 199.85 mm^2^ (100%), the expected BCA in the BSSO group decreased to 1344.70 ± 287.23 mm^2^ (72.3%) after the 8-mm setback and 1164.00 ± 295.50 mm^2^ (62.6%) after the 8-mm advancement. In contrast, the achieved BCA was only 413.52 ± 169.52 mm^2^ (22.2%) after the 8-mm setback and 391.38 ± 189.01 mm^2^ (21.0%) after the 8-mm advancement.

In the HSSO group, significant differences (*p* < 0.023) were only found between the reference BCA of 563.24 ± 96.18 mm^2^ (100%) and the expected BCA after the 8-mm setback (349.11 ± 98.42 mm^2^, 62.0%) and 8-mm advancement (352.58 ± 96.55 mm^2^, 62.6%). The achieved BCA was 278.03 ± 97.65 mm^2^ (49.4%) after the 8-mm setback and 229.37 ± 75.90 mm^2^ (40.7%) after the 8-mm advancement.

Regarding ICD and OCD, significant differences between the expected and achieved intercondylar distances were found for almost all statistical comparisons (Table 2). Both intercondylar distances underwent less change in reality than the virtual planning suggested. The only exceptions were the ICD and OCD results after the 4-mm setback in the BSSO group (expected: -1.07 ± 0.63 mm vs. achieved − 0.96 ± 0.44 mm, *p* = 0.926) and the HSSO group (expected: -1.23 ± 0.97 mm vs. achieved − 0.94 ± 0.63 mm, *p* = 0.541).

For the surgical planning, the expected values demonstrated higher increases in ICD and OCD in the HSSO than the BSSO group. In this context, significant differences between the techniques were found for ICD after the maximum mandibular setback of 8 mm (HSSO: -5.76 ± 1.07 mm vs. BSSO: -4.28 ± 1.58 mm, *p* = 0.037) and the maximum mandibular advancement of 8 mm (HSSO: 4.51 ± 0.73 mm vs. BSSO: 3.25 ± 1.17 mm, *p* = 0.012) as well for OCD after the 4-mm advancement (HSSO: 4.08 ± 0.39 mm vs. BSSO: 3.07 ± 1.28 mm, *p* = 0.041) and the 8-mm advancement (HSSO: 6.56 ± 0.72 mm vs. BSSO: 4.94 ± 1.47 mm, *p* = 0.003). However, no significant differences were found in the condylar position after the surgical procedures (*p* ≥ 0.271) (Fig. 7).

Concerning the amount of displacement, in most cases, the expected values verified significant increases for both intercondylar distances between the 4-mm and 8-mm advancements, and significant decreases were noticed after the 4-mm and 8-mm setbacks (Fig. 7). However, no statistically significant differences were found for ICD as a result of the mandibular setbacks in the BSSO group (-4 mm: 2.88 ± 1.44 mm vs. -8 mm: -4.28 ± 1.58 mm, *p* = 0.118). The results for the achieved changes were similar. However, they were not significant in the context of the mandibular setbacks for ICD (4 mm: -0.74 ± 0.56 mm vs. -8 mm: -1.28 ± 0.84 mm, *p* = 0.138) or OCD (-4 mm: -0.94 ± 0.63 mm vs. -8 mm: -1.58 ± 0.84 mm, *p* = 0.060).

## Discussion

Numerous studies have explored the use of HSSO on the ascending ramus of the mandible to facilitate mandibular movement in orthognathic surgery [7, 8, 11, 13, 16–18]. These studies have highlighted significant challenges related to determining the optimal location and orientation of the osteotomy cut and effectively securing bone segments to prevent injuries to the inferior alveolar nerve and temporomandibular joint (TMJ) disorders. These challenges need to be addressed in order to enhance the long-term stability of skeletal movements and promote optimal bone healing. In addition, the potential development of postoperative TMJ disorders has been examined, [19, 20] and the risk has been found to be considerably higher in patients who had preoperative TMJ dysfunction compared to those without such dysfunction [21]. 

The literature contains less information about the BCA in the context of surgical mandibular displacement. In this context, it is known that BCA is a plate loading factor and that plate failure can be associated with a small contact area [22]. Recently, Chen et al. [23] studied the BCA with regard to the mandibular sagittal split of large maxillomandibular advancements for obstructive sleep apnea. However, the study indicated that an optimal passive overlap of condylar and dentate segments was achieved in 91.9% of cases. Previous preclinical studies have examined the influence of the splitting technique and the extent of the displacement distance on the possible BCA between the distal and proximal segments as well as the position of the condyles [13, 24]. These studies suggested that a larger bony contact should be expected at any mandibular displacement distance for BSSO compared to HSSO and that both splitting techniques seem to result in similar changes in TMJ position [13, 24]. In this context, the percentage of BCA in relation to the initial situation appeared to be influenced more by the splitting technique than the displacement distance [13]. In contrast, the amount of displacement seems to have more influence than the osteotomy on the TMJ position [13, 24]. 

The present study employed established study designs were used [13, 24]. So, a research approach involving fresh human cadaver heads was adopted to achieve outcomes that closely resembled clinical scenarios. While employing fresh-body donors is considered more advantageous than utilizing fixed anatomical specimens and closely aligns with clinical reality [25, 26], there are certain limitations in the study design that impede the generalization of results. Firstly, potential discrepancies in tissue behavior may arise from the dehydration of bone and soft tissue, as well as the absence of perfusion. This impacts specific muscles in the localized area, exerting muscle pull and tension on the distal or proximal bony segments in living patients during corresponding surgical procedures. These forces influence segment repositioning and the fixation of osteosynthesis, potentially complicating the process. Thus, the operation on the body donor is more easily manageable, also due to the absence of bleeding. Secondly, the body donors utilized were of advanced age, whereas orthognathic surgery is typically conducted on young to middle-aged adults [27]. Finally, the transferability of the present study’s results to clinical reality is constrained by the absence of subjects with sagittal skeletal class II or III anomalies requiring surgery. The unique anatomical conditions linked to these skeletal malocclusions might impact the analyzed parameters. Consequently, it is essential to view the present results as an approximation of clinical reality.

This investigation was conducted as a continuation of a computer-simulated study [13] and had the primary aim of evaluating the BCA between the proximal and distal segments. Furthermore, it aimed to analyze the spatial changes in the positioning of the condyles in relation to each other. Specifically, this study sought to compare the outcomes of HSSO and BSSO procedures, which involved varying degrees of mandibular advancement and setback, by employing CBCT imaging techniques. However, the maxillary occlusal plane was not considered in the operative planning in this investigation, even though it could affect mandibular movement in vertical directions. But, these potential alterations of the condyle should be rated rather marginal [28]. 

The expected BCA values in the present study were similar to those of a previous investigation and demonstrated a linear contact decrease between the respective segments in the context of mandibular forward and backward displacement [13]. So, the simulated BCA in the HSSO group decreased to 67.05% after the 8-mm setback and 66.61% after the 8-mm advancement; in the BSSO group, the simulated BCA decreased to 82.72% after the 8-mm setback and 74.16% after the 8-mm advancement [13]. The present study yielded similar findings. After 8-mm setback and 8-mm advancement, BCA decreased to 62% and 62.6%, respectively, in the HSSO group and to 72% and 62.6%, respectively, in the BSSO group.

In the HSSO group, BCA was initially was relatively low, but, compared to the BSSO group, it decreased less with increasing displacement (4-mm vs. 8-mm displacements). In the BSSO group, increasing mandibular displacement led to statistically significant decreases in the expected BCA. Meanwhile, statistically significant differences were only found in the HSSO group when comparing the maximum advancement or setback with the reference BCA. Generally, the expected BCA was significantly lower in the HSSO group compared to the BSSO group at all times.

In the HSSO and BSSO group the achieved and expected BCA decreased during the increasing mandibular displacement. However, the achieved BCA after HSSO appeared to be significantly lower than after BSSO in all cases. Compared to the reference BCA, the achieved BCA decreased after the 8-mm setback and the 8-mm advancement to 49.4% and 40.7%, respectively, in the HSSO group and by 22.2% and 21.0%, respectively, in the BSSO group. With regard to the mean values, no statistically significant differences were observed between the groups at the 8-mm setback or the 8-mm advancement. Thus, the BCA seems to be an equivalent in the cases of maximum displacement.

Research findings have indicated that biomechanical stress is imposed on the TMJ during sagittal splitting, especially in the case of the BSSO technique, due to rotational forces that can cause condylar pressure within its fossa [29]. Additionally, manual repositioning of the proximal segment followed by osteosynthesis can lead to compression of the disc-condyle complex.^33^ In this context, previous studies have identified varying incidences of newly developed TMJ complaints, ranging from 0 to 4.3% related to HSSO [30] and 16.3% related to BSSO [31]. Seifert et al. [12] observed relatively low postoperative TMJ complaint rates, specifically, 2.3% in the HSSO group and 5.5% in the BSSO group. They attributed this discrepancy between the groups to the exclusion of patients from the study who had preexisting TMJ complaints before undergoing surgery.

Seeberger et al. [16] described possible changes in the intercondylar distance in this context, reporting a mean increase of 0.31 mm in the intercondylar distance in all patients, regardless of whether they had a mandibular advancement or setback with or without an additional cranial maxillary impaction. These findings were in contrast to Möhlhenrich et al.’s preclinical investigation results [13, 24]. The computer-simulated study revealed a statistically significant increase in the intercondylar distance during mandibular advancement and a reduction during mandibular setback [13]. However, no significant differences between the splitting groups were observed. In a further cadaveric investigation, a more detailed analysis of the TMJ changes revealed that, although the inner and outer intercondylar distances increased with mandibular advancement and decreased after setback, no statistically significant differences were observed between the two osteotomy methods [24]. Even though the outer intercondylar distance was relatively larger in cases of mandibular advancement based on BSSO and smaller in those based on HSSO, these differences did not reach statistical significance. This suggests rotational movement of the proximal segment, particularly in the axial plane, leading to a significant reduction in the intercondylar angle between the proximal segments in the HSSO group.

In the present study, the primary focus was on BCA because TMJ alteration was intensively examined in a previous cadaveric study [24]. However, because in this investigation the intercondylar distances were only determined within the transversal plan in the CBCT scan, which could indicate the possibility of inaccuracies with respect to this parameter, the direct intercondylar distances in the 3D reconstruction were measured in the present investigation.

The expected inner intercondylar distance changes in the present study were similar to those in a previous study. With regard to the 8-mm advancement, increases of 5.45 mm and 5.05 mm were found in the HSSO and BSSO groups, respectively [13]. Regarding the 8-mm setback, the mean decreases were 3.95 mm in the HSSO groups and 5.63 mm in the BSSO group. In comparison, the present study revealed increases in the HSSO and BSSO groups of 4.51 mm and 3.25 mm, respectively, for advancement and decreases of 5.76 mm and 4.28 mm, respectively, for setback. Consequently, the present results were not unexpected. Comparing the expected and achieved measurements revealed that, for both split techniques, the intercondylar distance increased in the case of mandibular advancement and decreased in the case of mandibular setback. However, in contrast to virtual planning, as anticipated, the achieved changes were statistically significantly smaller. Exceptions to this were the ICD and OCD results for the 4-mm setback. The achieved intercondylar distance changes confirmed the results of the previous cadaver study [24]. For example, with regard to ICD, the mean decreases after the 8-mm setback were 2.57 mm and 1.58 mm, respectively, for the HSSO and BSSO groups in the present study and 1.81 mm and 1.6 mm, respectively, for the HSSO and BSSO groups in the other study [24]. Regarding the 8-mm advancement, the changes were 2.07 mm and 1.7 mm, respectively, for the HSSO and BSSO groups in the present study and 2.12 mm and 2.14, respectively, for the HSSO and BSSO groups in the other study. Furthermore, in the present study, there was a slight difference between the inner and outer intercondylar distance results, which suggested a minor rotation of the proximal segment.

## Conclusion

The present study showed that the expected changes in bone contact area and intercondylar distance were not clinically realizable. Both, BCA and ICD/OCD seems to be less affected in reality than in virtual planning. Especially, the achieved BCA after BSSO seemed to be comparable to that of HSSO, especially for larger displacement distances and is maybe less important than previously assumed.

## Data Availability

The datasets used and/or analyzed during the current study are available from the corresponding author upon reasonable request.
